# Gelatin/Lignin Hydrogel Loaded with Mesenchymal Stem Cell-Derived Exosomes Enriched in Microrna-185 Inhibits Progression of Oral Cancer

**DOI:** 10.3390/pharmaceutics18030363

**Published:** 2026-03-14

**Authors:** Meitong Liu, Kai Wang, Can Zeng, Yijiang Jia, Jiaqi Wang, Ayijiang Taledaohan, Yuji Wang, Xiaobing Guan

**Affiliations:** 1 School of Stomatology, Capital Medical University, Beijing 100070, China; aryalmtong@163.com (M.L.); zengcandx@163.com (C.Z.); jiaqiwang5923@163.com (J.W.); 2 Department of Medicinal Chemistry, School of Pharmaceutical Sciences, Capital Medical University, Beijing 100069, China; ito1g2@163.com (K.W.); jiayijiang@ccmu.edu.cn (Y.J.); 3 Beijing Key Laboratory of Drug Innovation for Neuro-Oncology, Beijing Engineering Research Center of Targeted Drugs and Cell Therapy for CNS Tumors, Laboratory for Clinical Medicine, Capital Medical University, Beijing 100069, China; 4 Department of Emergency Medicine, University of California San Diego, San Diego, CA 92903, USA

**Keywords:** hydrogel, MSC-derived EVs, microRNA-185, OSCC, drug delivery

## Abstract

**Purpose:** Due to the lack of effective local therapeutic strategies for oral squamous cell carcinoma (OSCC), this study aimed to develop a novel gelatin/lignin hydrogel loaded with mesenchymal stem cell (MSC)-derived exosomes enriched in microRNA-185 (miR-185 EV) for intraoral delivery, followed by systematic evaluation of its therapeutic efficacy and underlying molecular mechanisms. **Materials and Methods:** The gelatin/lignin hydrogel was prepared and subsequently loaded with miR-185 EV. The physicochemical properties of the hydrogel, including microstructure, swelling behavior, chemical composition, and rheological characteristics, were systematically evaluated. Next, the stability, viscosity, biocompatibility, and exosome release kinetics of the hydrogel were further assessed. A 4-nitroquinoline-1-oxide (4NQO)-induced mouse tongue carcinogenesis model was established to assess the in vivo antitumor activity of the hydrogel via intraoral administration. Moreover, a proteomic analysis was conducted to investigate the molecular mechanisms of miR-185 EV on OSCC. **Results:** The miR-185 EV-loaded gelatin/lignin hydrogel exhibited favorable physicochemical properties, stability, and biocompatibility while prolonging the tissue retention time of miR-185 EV. In vivo antitumor efficacy experiments showed that the miR-185 EV-loaded hydrogel significantly inhibited tumor occurrence and alleviated epithelial dysplasia. Immunohistochemical analyses revealed significant suppression of tumor proliferation and epithelial–mesenchymal transition (EMT) of the hydrogel. Proteomic analysis indicated that miR-185 EV suppressed OSCC progression by downregulating interleukin-1β (IL-1β), consequently inhibiting the NF-κB signaling pathway. **Conclusion:** The findings demonstrate the successful development of the miR-185 EV-loaded gelatin/lignin hydrogel that represents an effective nanomedicine platform for intraoral drug delivery, providing a promising strategy for the clinical treatment of OSCC.

## 1. Introduction

Oral squamous cell carcinoma (OSCC) is the most common malignant tumor in the head and neck region, accounting for approximately 90% of cancers in this area [1]. This disease carries an extremely poor prognosis, with a five-year survival rate of only 50% [2]. According to global epidemiological statistics, annual new cases of OSCC reach 377,713, with related deaths as high as 177,757 [3], reflecting its considerable global health burden. The current treatment strategies for OSCC primarily involve surgical resection combined with radiotherapy and chemotherapy [4]. Nonetheless, surgical procedures can cause irreversible damage to maxillofacial aesthetics and oral functions, whereas radiotherapy and chemotherapy frequently induce adverse effects such as persistent fatigue, nausea, and immunosuppression, collectively undermining treatment adherence and diminishing patients’ quality of life [5,6]. Consequently, the development of effective early-intervention strategies represents a crucial objective for improving clinical outcomes in OSCC.

In this context, microRNAs (miRNAs) have emerged as promising therapeutic targets. These small non-coding RNA molecules regulate gene expression post-transcriptionally and are deeply implicated in oncogenesis [7,8]. Among them, microRNA-185 (miR-185) has been identified as a tumor suppressor, with its expression levels inversely correlated with cancer progression. Notably, experimental evidence confirms that upregulating miR-185 expression effectively inhibits OSCC progression [9], highlighting its potential as a therapeutic target.

However, naked extracellular miRNAs exhibit limited therapeutic durability due to structural instability and rapid enzymatic degradation, significantly restricting their sustained efficacy at lesion sites [10]. Exosomes, as naturally secreted extracellular vesicles, present a promising delivery solution [11]. These nano-scale particles (30–150 nm in diameter) possess a bilayer lipid membrane that enables stable encapsulation of bioactive cargo, including proteins, mRNAs, and miRNAs [12]. Particularly, MSC-derived exosomes display low immunogenicity, minimal toxicity, and excellent biocompatibility, making them ideal nanocarriers for therapeutic delivery [13]. Our previous studies have demonstrated that exosomes with high expression of miR-185 modulate inflammation and promote apoptosis via Akt/NF-κB and Akt/caspase-9 pathways, thereby impeding the progression from oral potentially malignant disorders (OPMDs) to OSCC [14].

Despite showing promising therapeutic potential, the clinical application of miR-185 EV in the oral cavity remains challenging. The flushing action of saliva and mechanical forces in the oral environment prevent sustained retention of miR-185 EV at lesion sites, resulting in uneven local drug concentration and compromised therapeutic efficacy [15,16]. Therefore, it is imperative to optimize local delivery strategies and design a simple, efficient system for clinical exosome therapy.

Hydrogels, as hydrophilic polymeric materials with a three-dimensional network structure [17], have emerged as ideal carriers owing to their tunable physical properties, exceptional biocompatibility, and controllable degradability [18]. Hydrogels not only enhance mucoadhesive properties for targeted drug localization and sustained release, but also protect drugs from salivary washout and mechanical forces in the oral environment, thereby significantly improving drug bioavailability [19,20]. The application of hydrogels in the treatment of oral disease is well documented. For example, an adhesive and lipophilic hydrogel was developed to deliver rapamycin for preventing the malignant transformation of oral leukoplakia [21]. Additionally, an intelligent gating hollow mesoporous silica hydrogel was designed for targeted periodontitis therapy [22]. Tissue engineering studies further demonstrate that exosome-loaded hydrogels can shield exosomes from immune clearance in vivo, prolong their tissue retention time and enhance their therapeutic efficacy [23]. Considerable evidence has confirmed the therapeutic potential of exosome-loaded hydrogels in oral disease treatment [24,25,26].

Among various hydrogel materials, gelatin/lignin hydrogels have garnered growing interest due to their unique advantages. Both gelatin and lignin are naturally derived polymers with wide availability and strong renewability. Gelatin/lignin hydrogels exhibit good biocompatibility and biodegradability, and their straightforward synthesis further supports the potential for biomedical applications [27,28]. Although gelatin/lignin hydrogels have shown therapeutic promise in wound healing applications [29,30], their utilization in oral diseases, particularly as exosome-loaded delivery systems, remains unexplored.

Building on this background, we sought to design a gelatin/lignin hydrogel loaded with miR-185 EV to overcome the limitations of local oral drug delivery and provide a novel therapeutic strategy for early intervention in OSCC.

## 2. Materials and Methods

### 2.1. Materials

Gelatin and lignin were provided by Macklin Biochemical Co., Ltd. (Shanghai, China) NaOH and EDC were supplied by Aladdin Biochemical Technology Co., Ltd. (Shanghai, China) MSC-derived exosomes transfected with miR-185 mimics were provided by Jieteng Bio Co., Ltd. (Beijing, China) Cell Counting Kit-8 (CCK-8) was purchased from Xinsaimei Biotechnology Co., Ltd. (Suzhou, China) The BCA protein assay kit was purchased from Beyotime Biotechnology Co., Ltd. (Shanghai, China) 4NQO was purchased from Sigma-Aldrich Co. LLC (St. Louis, MO, USA). The human oral keratinocyte cells (HOK) and human OSCC cells (Cal27) were obtained from American Type Culture Collection (ATCC) (Manassas, VA, USA).

### 2.2. Characterization of miR-185 EV

The protein concentration of exosomes was quantified using a BCA Protein Assay Kit. The particle size distribution of exosomes was identified by a nanoflow cytometer (N30 Nanoflow Analyzer, NanoFCM Inc., Xiamen, China). Western blotting was conducted to detect specific surface markers of exosomes, including CD9, CD63 and Calnexin. Transmission electron microscopy (TEM; JEM-2100, JEOL Ltd., Tokyo, Japan) was performed to observe the morphology of the exosomes.

### 2.3. Western Blotting

Protein was extracted from exosomes with RIPA lysis buffer (Beyotime, Shanghai, China) containing a protease inhibitor cocktail (Soleibao, Beijing, China). Protein samples with equal concentrations were separated by SDS–PAGE and transferred onto PVDF membranes (Bio-Rad, Hercules, CA, USA). The membranes were blocked in 5% skim milk and incubated overnight at 4 °C with primary antibodies against CD9 (ABclonal, Wuhan, China, A1703, 1:1000), CD63 (ABclonal, Wuhan, China, A19023, 1:1000), and Calnexin (Wanlei, Shenyang, China, WL03062, 1:1000). Subsequently, the membranes were treated with HRP-conjugated goat anti-rabbit IgG (Xinsaimei, Suzhou, China, P8002, 1:5000) for 1 h at room temperature, and the protein bands were detected using an enhanced chemiluminescence system (Millipore, MA, USA).

### 2.4. Preparation of miR-185 EV-Loaded Gelatin/Lignin Hydrogel

The gelatin/lignin hydrogel was synthesized according to the established protocols described in the literature [28]. First, 3.5 g of lignin was added to 100 mL of 5% *w*/*w* NaOH and stirred magnetically at 60 °C overnight to ensure complete dissolution. Then, 10 g of gelatin was dissolved in 100 mL of deionized water and stirred magnetically at 80 °C overnight. The two solutions were mixed at a certain ratio to obtain a gelatin/lignin hydrogel containing 1% lignin. The prepared solution was poured into a Petri dish and left to stand for 1 h to achieve complete solidification. The solidified hydrogel was immersed in 10 mL of the crosslinking agent EDC and kept at room temperature overnight. Afterward, the samples were washed repeatedly with deionized water, frozen at −80 °C overnight, and subsequently freeze-dried for storage. The freeze-dried hydrogel was dissolved in PBS at 37 °C at a ratio of 1:4.8 (*w*/*v*), followed by the addition of miR-185 EV at a ratio of 1:4.2 (*w*/*v*) on ice. The components were mixed thoroughly to achieve a final total ratio of 1:9 (*w*/*v*).

### 2.5. Scanning Electron Microscopy (SEM)

The prepared hydrogel samples were frozen at −80 °C and subsequently dehydrated in a vacuum freeze dryer for 48 h. The freeze-dried hydrogels were then fractured in liquid nitrogen to obtain cross-sections and sputter-coated with gold (Quorum SC7620 sputtering coater, Quorum Technologies Ltd., Lewes, UK). The morphological characteristics of the samples were examined using a Czech TESCAN MIRA LMS scanning electron microscope, with an accelerating voltage of 3 kV during imaging.

### 2.6. Fourier-Transform Infrared Spectroscopy (FTIR)

The freeze-dried hydrogels were ground into powder. The formation of chemical bonds and functional groups in the gelatin hydrogel, gelatin/lignin hydrogel, and miR-185 EV-loaded gelatin/lignin hydrogel was analyzed using an FTIR-iS5 infrared spectrometer (Thermo Fisher, Waltham, MA, USA). Changes in the characteristic absorption bands were used to determine these structural features. The spectral scanning range was 400–4000 cm^−1^ with a resolution of 4 cm^−1^.

### 2.7. Zeta Potential and Hydrodynamic Diameter Test

For dynamic light scattering (DLS) and zeta potential analysis, the gelatin/lignin hydrogel was swollen in PBS at a concentration of 1 mg/mL and then briefly treated with low-power sonication to obtain a uniform dispersion. The zeta potential and hydrodynamic diameter of the dispersed particles were determined using a Zetasizer Nano ZS (Malvern Instruments, Malvern, UK)

### 2.8. Stability Test

The gelatin/lignin hydrogel was immersed in PBS (pH 7.4) and stored at 4 °C. Its macroscopic stability (including structural integrity and any visual changes) was assessed by photographic documentation on days 1, 7, 14, 21, and 28.

### 2.9. Swelling Test

The gelatin/lignin hydrogel samples were prepared, and their initial dry weight (Wd) was recorded. The samples were then immersed in PBS. At designated time points, they were removed and gently blotted with filter paper to eliminate excess surface liquid. The swollen weight (Ws) was subsequently measured. The swelling ratio was calculated according to the following formula: Swelling ratio = (Ws − Wd)/Wd × 100%.

### 2.10. Rheological Analysis

The rheological properties of the gelatin/lignin hydrogel and miR-185 EV-loaded gelatin/lignin hydrogel were detected using the MCR 302 rheometer (Anton Paar GmbH, Graz, Austria). The prepared hydrogels were shaped into disks (25 mm diameter, 1–2 mm thickness) and tested in rotor oscillation mode for 60 min. The storage modulus (G’) and loss modulus (G”) were recorded at a constant strain of 0.1% and a constant frequency of 1 Hz.

### 2.11. Adhesive Test

The adhesive properties of the gelatin/lignin hydrogel and miR-185 EV-loaded gelatin/lignin hydrogel were tested using a microcomputer-controlled universal testing machine (CMT4503, MTS Industrial Systems, Shenzhen, China). Specifically, the hydrogels were cut into thin films (25–26 mm length × 20–21 mm width), placed between two pieces of fresh porcine skin tissue, and rolled back and forth three times with a 2 kg standard rubber roller. Then, the lap-shear test was performed with a 10 N load cell. The adhesive strength (kPa) was calculated by dividing the maximum load (kN) by the contact area (m^2^).

### 2.12. Exosome Release Kinetics from miR-185 EV-Loaded Gelatin/Lignin Hydrogel

A total of 100 μL miR-185 EV-loaded gelatin/lignin hydrogel was placed in the upper chamber of a 24-well Transwell plate, and 400 μL PBS was added to the lower chamber. The plate was incubated at 37 °C, and at predetermined time points, the supernatant was collected and replaced with an equal volume of fresh PBS. A standard curve was plotted using the BCA Protein Assay Kit to quantify the concentration of released exosomes. The cumulative exosome release kinetics were calculated and plotted as a release curve.

### 2.13. Establishment of 4NQO-Induced Mouse Tongue Carcinogenesis Model

All animal experiments were approved by the Animal Ethical and Welfare Committee of Beijing Stomatological Hospital (Approval No. KQYY-202309-008). Thirty-five male C57BL/6 mice (6 weeks, mean weight 20 g) were purchased from the SPF Biotechnology Co., Ltd. (Beijing, China). All the animals obtained for the study were included in the experiment after acclimatization. No animals were excluded or died during the experiment. Five mice without any management were considered the control group. The remaining thirty mice were provided with water containing 100 mg/L 4NQO for 16 weeks, which were considered as the 4NQO-treated groups. From the 17th week, the 4NQO-treated groups were divided randomly into 6 groups: the PBS group (n = 5), the hydrogel group (n = 5), the 300 μg/mL EV group (n = 5), the 600 μg/mL EV group (n = 5), the 300 μg/mL EV + hydrogel group (n = 5), the 600 μg/mL EV + hydrogel group (n = 5). The mice were anesthetized with 2% isoflurane, and all treatments were administered by topical application in the oral cavity once a day. Body weights were recorded once a week, and the general conditions of the mice were monitored throughout the experiment. At the end of the 23rd week, the mice were euthanized, and their tongues were fixed in 10% formalin for histopathological and immunohistochemical staining. The subsequent analyses were performed by investigators blinded to the group allocation.

### 2.14. Tumor Occurrence

After completion of the animal experiment, the mice were sacrificed. The tumor occurrence in each group was recorded, including the total tumor count and the triaxial dimensions (length, width, and height) of each tumor. The mean value of these triaxial measurements was defined as the tumor radius (r), and the average tumor volume of each group was calculated according to the following formula: Tumor volume = 4/3πr^3^ (mm^3^).

### 2.15. Histopathological Analysis

The tongue tissues fixed in 10% formalin were dehydrated, embedded in paraffin, and sectioned. Hematoxylin and Eosin (H&E) staining was performed for histopathological analysis. The degree of epithelial dysplasia was assessed based on the architectural and cytological features in epithelium according to the 2022 WHO classification of head and neck tumors.

### 2.16. Immunohistochemistry Analysis

Immunohistochemical (IHC) staining was performed to evaluate the expression of proliferation and epithelial–mesenchymal transition markers. After deparaffinization and dehydration, the sections were incubated with primary antibodies at 4 °C overnight after blocking endogenous peroxidase. The primary antibodies were used as follows: anti-Ki67 (Servicebio, Wuhan, China, catalog no.gb121141, dilution 1:600), anti-E-cad (Servicebio, Wuhan, China, GB12083, 1:1000), and anti-Vimentin (Servicebio, Wuhan, China, GB111308, 1:1500). Next, the sections were incubated with secondary antibodies at room temperature for 50 min. The secondary antibodies were used as follows: HRP-conjugated goat anti-mouse IgG (Servicebio, Wuhan, China, GB23301, 1:200) for Ki67 and E-cad, and HRP-conjugated goat anti-rabbit IgG (Servicebio, Wuhan, China, GB23303, 1:200) for Vimentin. Finally, the slides were detected with DAB chromogen (Servicebio) and were stained with hematoxylin. All the sections were observed under the microscope (Olympus BX61, Japan) at 200× and 400× magnification, and three to five regions were randomly selected. The positive cell rate (%) of Ki67 and the positive area ratio (%) of E-cadherin and Vimentin were calculated using Fiji/ImageJ professional image analysis software (version 1.54p).

### 2.17. Biocompatibility of Hydrogel

After completion of the animal experiment, the major organs of the mice (the heart, liver, spleen, lung and kidney) were collected for H&E staining to evaluate the in vivo biocompatibility of the hydrogel. The HOK cells were cultured in Dulbecco’s Modified Eagle Medium (DMEM, Gibco, Grand Island, NY, USA) supplemented with 10% fetal bovine serum (FBS, Gibco, USA) and 1% penicillin/streptomycin (Gibco, Grand Island, NY, USA) at 37 °C in the humidified incubator containing 5% CO_2_. The CCK-8 kit was used to assess the cell proliferation and viability of the gelatin/lignin hydrogel and miR-185 EV-loaded gelatin/lignin hydrogel. To obtain hydrogel extracts, 10 mg of the dried hydrogel samples were soaked in 10 mL of culture medium for 24 h, and then sterilized through a 0.22 μm filter. The HOK cells were seeded into 24-well plates at a density of 5 × 10^4^ cells per well. After cell adhesion, the experimental group was treated with hydrogel extracts, while the control group received an equal volume of DMEM medium. After incubation for 24 h, CCK-8 reagent was added and allowed to react for 1 h. Subsequently, the optical density (OD) at 450 nm wavelength was measured by a microplate reader to calculate cell viability according to the following formula: Cell viability = (A_sample_ − A_blank_)/(A_control_ − A_blank_) × 100%. Where A_sample_ and A_control_ represent the absorbance of the hydrogel group and the control group, respectively, and A_blank_ represents the absorbance of the CCK-8 solution without cells.

### 2.18. Proteomics Analysis

The Cal27 cells were seeded in 6-well plates at a density of 5 × 10^5^ cells per well in 2 mL of DMEM medium (supplemented with 10% FBS and 1% penicillin/streptomycin) and cultured in a 37 °C CO_2_ incubator. The following day, after cell adhesion, the experimental group was treated with 200 μg/mL miR-185 EV. While the control group was treated with an equal volume of PBS. There were four replicates in each group. After 24 h of incubation, the cells were washed twice with PBS, collected using a cell scraper, and transferred into centrifuge tubes. The samples were centrifuged at 4 °C at 1000× *g* for 10 min to collect the cell pellets, which were stored in dry ice and sent to Shanghai OE Biotech Co., Ltd. (Shanghai, China) for proteomic data analysis. Proteins with a fold change (FC) ≥ 1.5 or FC ≤ 1/1.5 and *p*-value < 0.05 between the experimental and the control groups were identified as differentially expressed proteins (DEPs). DEPs were further used for Gene Ontology (GO) and Kyoto Encyclopedia of Genes and Genomes (KEGG) enrichment analysis. Protein–protein interaction (PPI) analysis was performed using the String.

### 2.19. Statistical Analysis

Data following a normal distribution were expressed as mean ± standard deviation (n ≥ 3). Normality was assessed using the Shapiro–Wilk test; if *p* > 0.05, the data were considered normally distributed. Homogeneity of variance was evaluated using the Brown-Forsythe test, and if *p* > 0.05, the data were regarded as having equal variances. For data that met both normality and homogeneity assumptions, one-way analysis of variance (ANOVA) was used to compare the total mean, and the Student’s *t*-test was applied for pairwise comparisons. For data that did not meet these assumptions, the Kruskal–Wallis test was used for analysis. Statistical analysis was conducted by using GraphPad Prism 9 software. Statistical significance was defined as * *p* < 0.05, ** *p* < 0.01, *** *p* < 0.001, and **** *p* < 0.0001; “ns” indicated no statistically significant difference.

## 3. Results

### 3.1. Preparation and Characterization of miR-185 EV-Loaded Gelatin/Lignin Hydrogel

**Figure 1:**
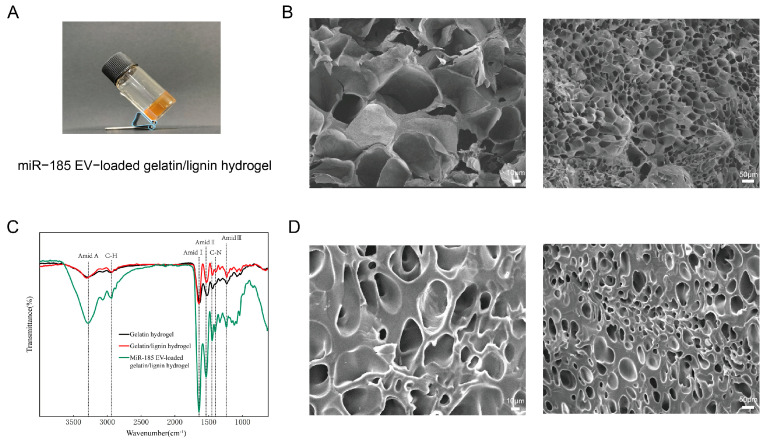
Fabrication and physicochemical characterization of the miR-185 EV-loaded gelatin/lignin hydrogel: (**A**) Macroscopic picture of the miR-185 EV-loaded gelatin/lignin hydrogel. (**B**) SEM image of gelatin/lignin hydrogel. Scale bar = 10 μm; 50 μm. (**C**) FTIR spectra of the gelatin hydrogel, gelatin/lignin hydrogel, and miR-185 EV-loaded gelatin/lignin hydrogel. (**D**) SEM image of the miR-185 EV-loaded gelatin/lignin hydrogel. Scale bar = 10 μm; 50 μm.

MiR-185 EVs were confirmed as exosomes by typical size distribution (43.1–148.3 nm in diameter, with an average size of 63.51 nm), Western blot detection of CD9 and CD63 with the absence of Calnexin, and TEM observation of typical spherical vesicles (Figure S1). Given the favorable properties of gelatin/lignin hydrogels, we adapted previously reported preparation methods to investigate formulations with different component ratios [28]. To explore a hydrogel with suitable viscosity for oral application, morphological observation and application tests were performed using exosome solutions mixed with freeze-dried gelatin/lignin hydrogels at various proportions. We found that when the lignin content was 1% in the gelatin/lignin hydrogel, and the exosome solutions and freeze-dried gelatin/lignin hydrogel were mixed at a ratio of 9:1, the prepared hydrogel exhibited appropriate viscosity for subsequent experiments. Using this formulation, we successfully prepared miR-185 EV-loaded gelatin/lignin hydrogel, which appeared as a yellow gel macroscopically (Figure 1A). The SEM imaging showed that the gelatin/lignin hydrogel presented an irregular, loose, and porous structure, which was suitable for encapsulating and releasing exosomes (Figure 1B). After loading exosomes, the pore diameter of the hydrogel slightly decreased (Figure 1D). The FTIR spectroscopy was performed to analyze the structural characteristics of the hydrogels, as shown in Figure 1C. By comparing the gelatin, gelatin/lignin, and miR-185 EV-loaded gelatin/lignin hydrogel, we observed that the incorporation of lignin reduced peak intensities of Amide II, C-N, and Amide III. After loading exosomes, the peak intensities corresponding to all amide bonds were enhanced.

### 3.2. Stability and Rheology of miR-185 EV-Loaded Gelatin/Lignin Hydrogel

**Figure 2:**
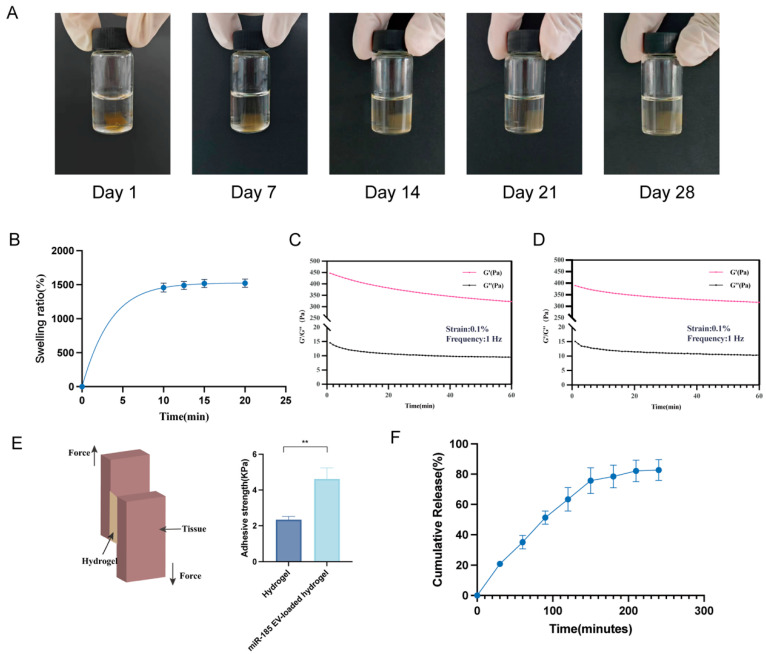
The miR-185 EV-loaded gelatin/lignin hydrogel exhibits favorable stability, rheology, adhesion, and sustained release properties: (**A**) Degradation time of the miR-185 EV-loaded gelatin/lignin hydrogel at 4 °C. (**B**) Swelling ratio of hydrogels in PBS (n = 3). (**C**) The time-dependent rheological properties of the gelatin/lignin hydrogel at a constant strain (0.1%) and frequency (1 Hz). (**D**) The time-dependent rheological properties of the miR-185 EV-loaded gelatin/lignin hydrogel at a constant strain (0.1%) and frequency (1 Hz). (**E**) Diagram of the lap-shear test and quantification of adhesive strength for the gelatin/lignin hydrogel and miR-185 EV-loaded gelatin/lignin hydrogel. (**F**) The release curves of miR-185 EV from the gelatin/lignin hydrogel (n = 3). Data represent mean values ± SD, ** *p* < 0.01.

The dispersed hydrogel particles exhibited a zeta potential of −8.86 ± 5.6 mV and an average hydrodynamic diameter of 408.2 ± 12.8 nm. The stability of the hydrogel was then assessed through a degradation time test. Morphological observations at days 1, 7, 14, 21, and 28 revealed no significant structural degradation (Figure 2A), indicating long-term stability at 4 °C. Swelling analysis showed that the gelatin/lignin hydrogel reached a stable equilibrium after 10 min (Figure 2B). Subsequently, rheological analysis was performed to evaluate the mechanical properties of the hydrogel. The G’ of the gelatin/lignin hydrogel exceeded its G″ (Figure 2C), and this trend remained unchanged after loading exosomes (Figure 2D), suggesting that exosome incorporation did not affect the hydrogel’s mechanical integrity. Additionally, the lap-shear test showed increased adhesive strength after exosome loading compared with the unloaded hydrogel (Figure 2E). Finally, exosome release kinetics revealed that the cumulative release reached a plateau at 240 min, with approximately 80–85% of the exosomes released. (Figure 2F).

### 3.3. MiR-185 EV-Loaded Gelatin/Lignin Hydrogel Improves General Health of 4NQO-Induced Mouse Tongue Carcinogenesis Model

**Figure 3:**
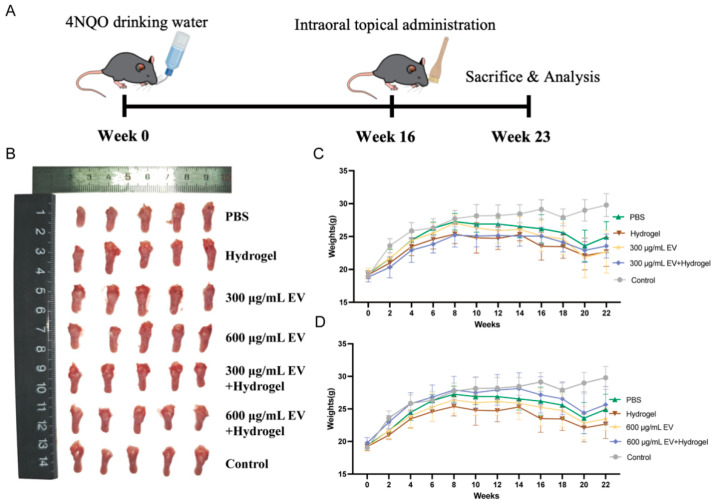
The miR-185 EV-loaded gelatin/lignin hydrogel improves general health in the 4NQO-induced mouse tongue carcinogenesis model: (**A**) A flow diagram showing the establishment of the 4NQO-induced mouse tongue carcinogenesis model. (**B**) Gross tumors and lesions in the mouse tongues at the end of the 23rd week. Scale ruler (mm). (**C**) Mouse body weight of PBS-, hydrogel-, 300 μg/mL EV-, 300 μg/mL EV + hydrogel-treated groups and control groups (n = 5 per group). (**D**) Mouse body weight of PBS-, hydrogel-, 600 μg/mL EV-, 600 μg/mL EV + hydrogel-treated groups and control groups (n = 5 per group). Data represent mean values ± SD.

To evaluate the therapeutic effect of the miR-185 EV-loaded gelatin/lignin hydrogel on OSCC, a 4NQO-induced mouse tongue carcinogenesis model was established. The hydrogel was administered via intraoral topical application, and tongue tissues were collected at the end of the experiment, as shown in the experiment flow diagram (Figure 3A). Macroscopic observation of the tongue mucosa at the 23rd week revealed that the control group exhibited normal tongue morphology with smooth, continuous mucosa and no visible proliferative lesions, while the 4NQO-treated groups displayed variably sized thorn-like proliferative lesions, with some larger masses resembling papillomas (Figure 3B). Throughout the experiment, the mice in the exosome and exosome–hydrogel group demonstrated improved activity levels compared with those in the PBS and hydrogel group. Body weight analysis showed a gradual increase in all the experimental groups from 0 to 14 weeks, followed by a progressive decline from the 14th week. Notably, the 300 μg/mL EV + hydrogel group exhibited a slower rate of weight loss than the 300 μg/mL EV group (Figure 3C). Furthermore, the 600 μg/mL EV + hydrogel group showed better body weight maintenance than other experimental groups throughout the experiment (Figure 3D). In conclusion, the results indicate that miR-185 EV-loaded gelatin/lignin hydrogel improves the general conditions of the 4NQO-induced mouse tongue carcinogenesis model.

### 3.4. MiR-185 EV-Loaded Gelatin/Lignin Hydrogel Suppresses Tumor Occurrence and Reduces Epithelial Dysplasia

**Figure 4:**
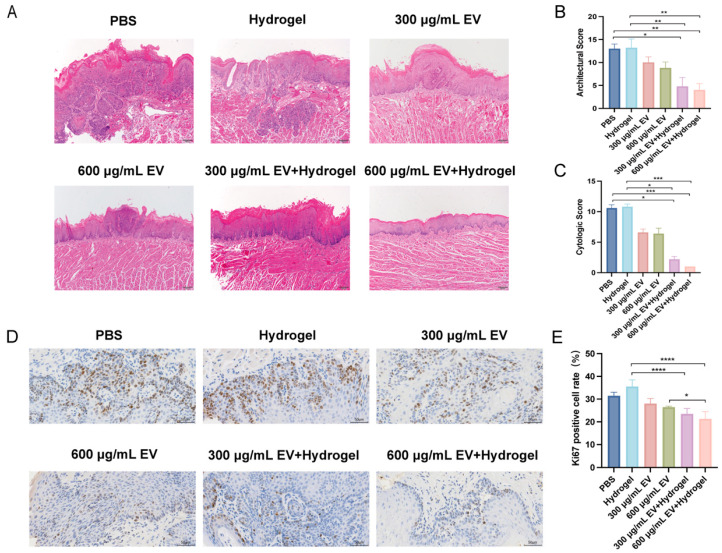
The miR-185 EV-loaded gelatin/lignin hydrogel suppresses epithelial dysplasia and tumor proliferation in vivo: (**A**) Histological H&E-stained sections of mouse tongues from each group are shown. Scale bar = 100 μm. (**B**,**C**) Pathological scores for oral epithelial dysplasia, including architectural and cytological scores of each group (n = 5 per group). (**D**,**E**) Ki67 was measured in the tongue tissues by IHC, and the percentage of Ki67-positive cells was analyzed in each group (n = 5 per group). Scale bar = 50 μm. Data represent mean values ± SD, * *p* < 0.05, ** *p* < 0.01, *** *p* < 0.001, **** *p* < 0.0001.

In the 4NQO-induced mouse tongue carcinogenesis model, macroscopic tumor analysis revealed that both the exosome group and exosome–hydrogel group exhibited reduced average tumor number and volume compared with the PBS group and hydrogel group. Statistical analyses showed that the 600 μg/mL EV + hydrogel group exhibited an average tumor number of 1.60 ± 0.55, which was significantly lower than that of the PBS and hydrogel groups (*p* < 0.05). In addition, the average tumor volume was significantly reduced to 1.86 ± 0.68 mm^3^ compared with the PBS group (*p* < 0.05; Table 1). To further compare the degree of epithelial dysplasia among the groups, H&E staining was performed on the dorsal tongue mucosa. The results showed marked differences in epithelial dysplasia across groups: the PBS group and hydrogel group exhibited more severe dysplasia, whereas the exosome group and exosome–hydrogel group showed reduced levels of dysplasia (Figure 4A). Next, quantitative pathological scoring of epithelial dysplasia, including both architectural features and cytological features, was performed. As shown in Figure 4B,C, the 300 μg/mL EV + hydrogel group exhibited reduced architectural and cytological scores compared with the PBS and hydrogel groups (*p* < 0.05), whereas the 600 μg/mL EV + hydrogel group displayed significantly lower scores (*p* < 0.01, *p* < 0.001). These findings suggest that miR-185 EV-loaded gelatin/lignin hydrogel suppresses tumor occurrence and reduces epithelial dysplasia in the 4NQO-induced mouse tongue carcinogenesis model.

### 3.5. MiR-185 EV-Loaded Gelatin/Lignin Hydrogel Inhibits Tumor Cell Proliferation In Vivo

We next investigated the effect of the therapy on tumor cell proliferation in vivo by immunohistochemical staining for Ki-67. Representative images revealed a high density of Ki-67-positive nuclei in the proliferative lesions of the PBS group, which was visibly reduced following treatment (Figure 4D). Quantitative analysis confirmed a potent anti-proliferative effect. Both the low-dose and high-dose miR-185 EV-loaded hydrogel treatments resulted in a dramatic reduction in the Ki-67 positive cell rate compared to the blank hydrogel control (*p* < 0.0001, Figure 4E). Importantly, the efficacy was significantly enhanced by the hydrogel delivery system. The Ki-67 positive cell rate in the high-dose combination group (600 μg/mL EV + hydrogel) was significantly lower than that in the group receiving the same dose of miR-185 EV without the hydrogel (*p* < 0.05, Figure 4E). These results demonstrate that the miR-185 EV-loaded gelatin/lignin hydrogel effectively suppresses tumor cell proliferation in vivo, with superior activity achieved through hydrogel-mediated delivery.

### 3.6. MiR-185 EV-Loaded Gelatin/Lignin Hydrogel Suppresses the EMT In Vivo

**Figure 5:**
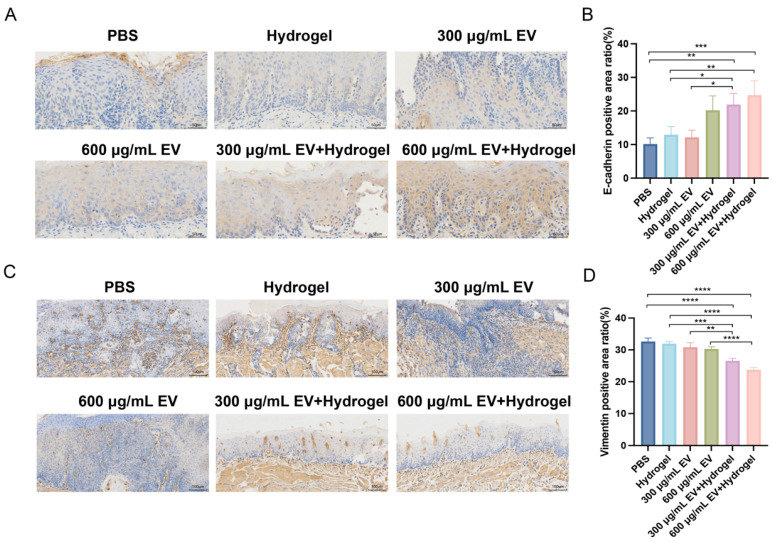
The hydrogel inhibits epithelial–mesenchymal transition (EMT) in vivo: (**A**,**B**) E-cadherin was measured in the tongue tissues by IHC, and the percentage of E-cadherin-positive areas was analyzed in each group (n = 3 per group). Scale bar = 50 μm. (**C**,**D**) Vimentin was measured in the tongue tissues by IHC, and the percentage of Vimentin-positive areas was analyzed in each group (n = 3 per group). Scale bar = 100 μm. Data represent mean values ± SD, * *p* < 0.05, ** *p* < 0.01, *** *p* < 0.001, **** *p* < 0.0001.

To investigate the effect of the miR-185 EV-loaded gelatin/lignin hydrogel on EMT in vivo, IHC staining was performed on the dorsal tongue mucosa to observe the expression of the epithelial marker E-cadherin and the mesenchymal marker Vimentin. As shown in Figure 5A,B, E-cadherin expression was significantly increased in both the 300 μg/mL EV + hydrogel and 600 μg/mL EV + hydrogel group compared with the PBS and hydrogel group (*p* < 0.05, *p* < 0.01, *p* < 0.001). Additionally, E-cadherin expression was significantly higher in the 300 μg/mL EV + hydrogel group than in the 300 μg/mL EV group (*p* < 0.05). As shown in Figure 5C,D, Vimentin expression was markedly decreased in the 300 μg/mL EV + hydrogel and 600 μg/mL EV + hydrogel group compared with the hydrogel group (*p* < 0.001, *p* < 0.0001). Moreover, Vimentin expression was significantly reduced in both the EV + hydrogel groups compared with their respective EV groups (*p* < 0.01, *p* < 0.0001). These results indicate that the miR-185 EV-loaded gelatin/lignin hydrogel effectively suppresses the EMT progression in vivo, with significantly enhanced therapeutic efficacy compared with miR-185 EV monotherapy.

### 3.7. MiR-185 EV-Loaded Gelatin/Lignin Hydrogel Exhibits Good Biocompatibility

**Figure 6:**
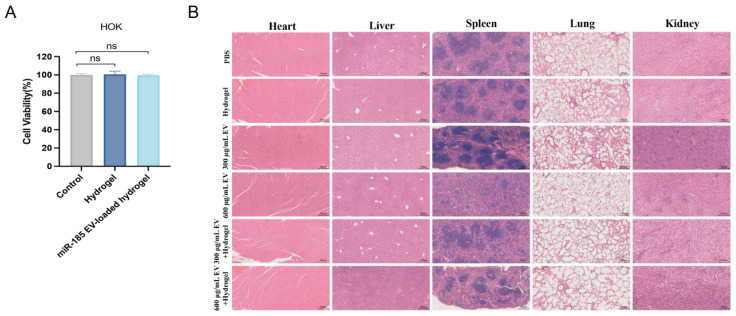
The hydrogel demonstrates excellent biocompatibility in vitro and in vivo: (**A**) Cell viability of HOK cells after incubating in hydrogel extracts for 24 h (n = 3 per group). (**B**) H&E staining of 4NQO-induced mouse major organs (heart, liver, spleen, lung, and kidney) after intraoral topical administration of hydrogel for 7 weeks. Scale bar = 200 μm. Data represent mean values ± SD, “ns” indicates no significant difference.

Before clinical application of oral therapeutics, it is essential to confirm the biocompatibility of the hydrogel. As shown in Figure 6A, both the hydrogel group and the miR-185 EV-loaded hydrogel group maintained a high cell survival rate of over 95%, showing no statistically significant cytotoxicity compared to the control group, indicating excellent cytocompatibility of the hydrogel.

To further evaluate the histocompatibility with major organs, H&E staining was performed on visceral tissues from the 4NQO-induced mouse tongue carcinogenesis model following intraoral hydrogel administration. As demonstrated in Figure 6B, no obvious tissue damage or inflammatory lesions were observed in any major organ. Taken together, these findings indicate that miR-185 EV-loaded gelatin/lignin hydrogel possesses good biocompatibility and holds promise for further clinical translation.

### 3.8. MiR-185 EV Inhibits NF-κB by Downregulating IL-1β

**Figure 7:**
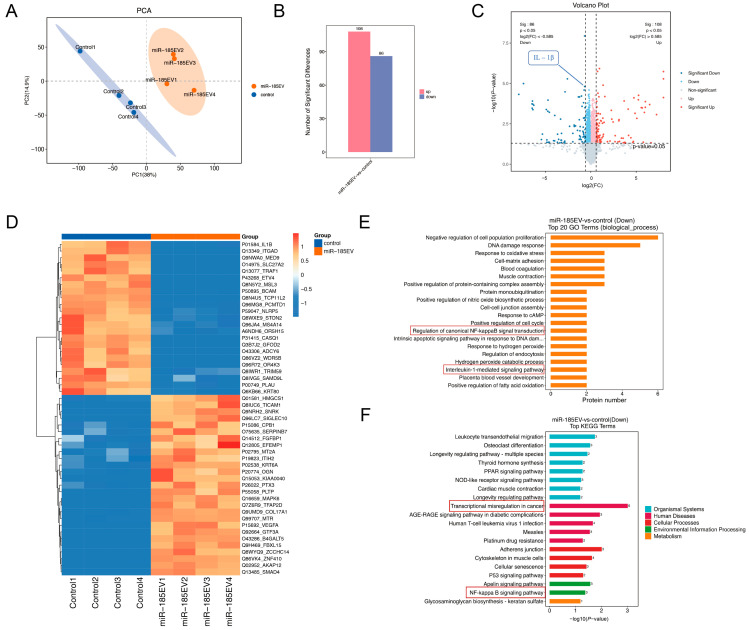
Proteomic analysis reveals IL-1β downregulation and NF-κB pathway inhibition by miR-185 EV: (**A**) PCA score plots between the control and miR-185 EV groups. (**B**) DEPs between the control and miR-185 EV groups. The red bar chart represents significantly upregulated proteins, and the blue bar chart represents significantly downregulated proteins. (**C**) Volcano Plot of differential proteins between control and miR-185 EV groups. The red dots represent significantly upregulated proteins, and the blue dots represent significantly downregulated proteins. (**D**) Heat map showing the top 50 highly expressed proteins. (**E**) Top GO-term categories for the different expressed proteins between the control and miR-185 EV groups. (**F**) Top KEGG-term categories for the different expressed proteins between the control and miR-185EV groups.

To further elucidate the therapeutic mechanism of miR-185 EV on OSCC, we performed quantitative proteomics analysis on Cal27 cells treated with miR-185 EV and PBS, with four biological replicates per group. A total of 8046 proteins were identified by liquid chromatography-tandem mass spectrometry (LC-MS/MS). The principal component analysis (PCA) score plot demonstrated high intra-group consistency and clear inter-group separation (Figure 7A). As shown in Figure 7B, a total of 194 differentially expressed proteins (DEPs) were identified (FC ≥ 1.5 or FC ≤ 1/1.5, *p* < 0.05), including 108 upregulated and 86 downregulated proteins. The volcano plot and cluster heatmap analyses revealed that IL-1β expression was significantly downregulated in the miR-185 EV group compared with the Control group (Figure 7C,D). GO enrichment analysis indicated that the DEPs were significantly enriched in the NF-κB and interleukin-1-mediated signaling pathway (Figure 7E). Moreover, KEGG enrichment analysis demonstrated marked enrichment in the NF-κB and transcriptional misregulation in cancer pathways (Figure 7F). These results are consistent with our previous findings in the dimethylbenzanthracene (DMBA) induced OPMD model, in which miR-185 EV suppressed IL-1β and NF-κB expression. Furthermore, KEGG pathway mapping confirmed that miR-185 EV inhibited NF-κB activation by downregulating IL-1β. The PPI network diagram (Figure S2) further analyzed and predicted the interacting proteins of IL-1β, providing new insights for subsequent mechanistic exploration and verification.

## 4. Discussion

In recent years, MSC-derived exosomes have attracted attention in disease therapy [31]. Our research group previously isolated and characterized MSC-derived miR-185 EV [14], thereby verifying the feasibility of localized miR-185 EV delivery for the treatment of OPMD in a hamster model. However, the clinical application of exosomes remains markedly constrained by inherent limitations, including insufficient targeting specificity, structural instability, and suboptimal intraoral biodistribution [32].

Hydrogel, as a hydrophilic three-dimensional network material, possesses strong water absorption capacity while maintaining structural integrity [33]. The synergistic effects of hydrogel-exosomes biological complexes enhance the tissue retention time and therapeutic effect of exosomes, playing an important role in the treatment of various diseases and demonstrating promising clinical therapeutic prospects [34,35].

Given the favorable properties of gelatin/lignin hydrogels, we successfully prepared a miR-185 EV-loaded gelatin/lignin hydrogel. The SEM analysis revealed a loose and porous structure, which is suitable for loading exosomes. The FTIR spectroscopy indicated that the addition of lignin markedly weakened the intensities of the Amide II, C-N, and Amide III peaks, suggesting multiple interactions between lignin functional groups and gelatin amino acid residues [36], while after loading exosomes, the intensities of the amide-related peaks increased, which is likely due to the presence of exosomal proteins. Additionally, hydrogel stability testing demonstrated that the hydrogel could be stably stored at 4 °C, supporting its feasibility for clinical storage and application. Notably, the oral cavity represents a dynamic environment characterized by salivary flow, enzymatic activity, and pH fluctuations. Gelatin-based hydrogels are susceptible to enzymatic cleavage [37], and degradation kinetics are influenced by material composition and crosslinking density [27]. Therefore, degradation in the oral cavity may be faster than under storage conditions. Future studies using simulated saliva with physiological collagenase levels are needed to better approximate intraoral conditions and evaluate degradation behavior. In addition to hydrogel degradation, the stability of the miR-185 within the hydrogel also warrants consideration. The present study primarily evaluated the macroscopic stability of the hydrogel under storage conditions and did not directly quantify the long-term stability of miR-185 within the hydrogel. Extracellular vesicles have been reported to protect encapsulated miRNAs from enzymatic degradation [38], and hydrogels are commonly used to maintain RNA stability and bioactivity during local delivery [39]. The therapeutic effects observed in the 4NQO-induced OSCC model suggest that the delivered miR-185 EV retained functional activity in vivo. Nevertheless, systematic evaluation of the long-term storage stability of miR-185 within the hydrogel will be necessary in future studies.

It is well established that incorporating lignin into gelatin enhances hydrogel swelling capacity by enlarging pore size and thinning pore walls, thereby accelerating water transmission rate [40,41]. Previous studies have reported that the strain sweep profiles of gelatin/lignin hydrogels exhibit a linear behavior up to 10%, suggesting complete crosslinking between gelatin and lignin to form a stable gel system [42]. Despite this, rheological analysis in our study confirmed that both gelatin/lignin and miR-185 EV-loaded gelatin/lignin hydrogels possessed good elasticity and adequate mechanical strength. Furthermore, the adhesive test and exosome release results suggested that the hydrogel exhibited adhesive properties and prolonged the local retention of exosomes. It should be noted that the lap-shear test was performed on porcine skin, which differs from oral mucosa in hydration levels and histological structure. Therefore, the measured adhesive strength provides a standardized mechanical comparison rather than a direct simulation of intraoral conditions [43]. Future studies will further evaluate adhesion on porcine buccal mucosa under wet or simulated-saliva conditions to better reflect the intraoral environment.

The 4NQO-induced mouse tongue carcinogenesis model is a well-established and reproducible experimental system for oral cancer induction, which can effectively simulate the human pathological progression from epithelial dysplasia to OSCC [44]. This model enables rigorous evaluation of therapeutic agents and mechanistic studies of oral carcinogenesis. To investigate the therapeutic effect of miR-185 EV-loaded gelatin/lignin hydrogel on OSCC, we established the 4NQO-induced mouse tongue carcinogenesis model. Experimental results revealed that the hydrogel significantly ameliorated disease progression and suppressed tumorigenesis in 4NQO-treated mice.

Epithelial–mesenchymal transition (EMT) is a critical mechanism in cancer progression, during which epithelial cells lose their epithelial characteristics and transform into mesenchymal cells [45]. This process is characterized by decreased expression of epithelial markers such as E-cadherin and increased expression of mesenchymal markers such as vimentin [46]. The PI3K/Akt signaling pathway has been extensively investigated as a potential therapeutic target in EMT regulation [47]. Studies in oral, colon, bladder, and breast cancers demonstrate that inhibition of the Akt pathway reverses EMT, thereby suppressing tumor growth and metastasis [48,49,50,51,52]. Our previous research has confirmed that miR-185 EV inhibits EMT by targeting Akt, thus inhibiting the early progression of OPMD to OSCC. In this study, we further detected that in the 4NQO-induced mouse tongue carcinogenesis model, miR-185 EV-loaded gelatin/lignin hydrogel significantly inhibited EMT and exhibited enhanced therapeutic efficacy compared with miR-185 EV alone. Collectively, these findings fully demonstrate that gelatin/lignin hydrogel serves as an effective delivery system for miR-185 EV, enhancing its therapeutic effect in oral cancer treatment.

Accumulating evidence highlights IL-1β as a pivotal pro-inflammatory cytokine involved in tumor pathogenesis [53], particularly in promoting malignant transformation and invasion in oral cancers [54]. Proteomics analysis in this study revealed significant downregulation of IL-1β protein expression, which is consistent with our previous findings, suggesting that miR-185 EV may inhibit OSCC by suppressing expression of IL-1β. The NF-κB signaling pathway is a central regulator of numerous physiological and pathological processes and plays essential roles in inflammatory modulation and immune homeostasis [55]. Building on our previous findings that miR-185 EV exerts anti-inflammatory effects through the Akt/NF-κB pathway, both GO and KEGG analyses in this study confirmed pronounced enrichment of the NF-κB pathway. Moreover, KEGG pathway mapping further indicated that miR-185 EV may inhibit NF-κB by downregulating IL-1β. Based on these findings, we performed a systematic analysis and prediction of the IL-1β-interacting proteins, providing new insights for exploring therapeutic mechanisms of miR-185 EV in OSCC. However, given the pleiotropic nature of miRNAs [56], it is likely that miR-185 EVs regulate multiple downstream targets and signaling pathways beyond those identified in this study [57], which warrants our deeper mechanistic exploration.

Collectively, miR-185 EV-loaded gelatin/lignin hydrogel provides an innovative therapeutic strategy for localized drug delivery in OSCC. This system holds considerable potential for synergistic integration with emerging technologies, including nanocarriers and 3D printing technology, to enhance targeting accuracy and improve therapeutic outcomes [58,59].

## 5. Conclusions

In this study, we successfully prepared miR-185 EV-loaded gelatin/lignin hydrogel. Material characterization indicated that the hydrogel exhibited a well-defined surface structure, sufficient elasticity and mechanical strength, and good stability and biocompatibility while prolonging the tissue retention time of miR-185 EV. In vivo experiments revealed that the hydrogel effectively inhibited OSCC progression by suppressing tumor cell proliferation and EMT in the 4NQO-induced mouse tongue carcinogenesis model. Furthermore, proteomic analysis demonstrated that miR-185 EV inhibited the NF-κB pathway through the downregulation of IL-1β, thereby providing a new direction for further mechanism exploration of OSCC. In summary, the hydrogel developed in this study shows considerable potential for clinical application and offers novel insights into local drug delivery strategies in OSCC treatment.

## Figures and Tables

**Table 1:** Tumor formation observed by naked eyes.

Group	Tumor Number	Tumor Volume (mm^3^)
PBS	3.80 ± 1.10	5.29 ± 0.98
Hydrogel	3.60 ± 0.55	4.09 ± 1.32
300 μg/mL EV	2.20 ± 0.45	3.87 ± 1.90
600 μg/mL EV	2.20 ± 0.84	2.96 ± 2.53
300 μg/mL EV + Hydrogel	2.00 ± 0.71	3.01 ± 1.16
600 μg/mL EV + Hydrogel	1.60 ± 0.55 *^,#^	1.86 ± 0.68 *

Data represent mean values ± SD, * *p* < 0.05 compared with the PBS group. ^#^ *p* < 0.05 compared with the hydrogel group.

## Data Availability

The mass spectrometry proteomics data have been deposited to the ProteomeXchange Consortium via the iProX partner repository with the dataset identifier PXD072609. The original contributions presented in this study are included in the article and Supplementary Materials. Further inquiries can be directed to the corresponding authors.

## Supplementary Materials

The following supporting information can be downloaded at: https://www.mdpi.com/article/10.3390/pharmaceutics18030363/s1, Figure S1: Characterization of miR-185 EV; Figure S2: The PPI of the top 25 differentially expressed proteins.
