# Supplementary material for: Gelatin/Lignin Hydrogel Loaded with Mesenchymal Stem Cell-Derived Exosomes Enriched in Microrna-185 Inhibits Progression of Oral Cancer
Source: Pharmaceutics. 2026 Mar 14;18(3):363. doi: 10.3390/pharmaceutics18030363 (PMC13030393; doi:10.3390/pharmaceutics18030363)
Supplement: Supplementary file 1 [file pharmaceutics-18-00363-s001.zip › pharmaceutics-4141965-Supplementary.pdf]

Supplementary Materials

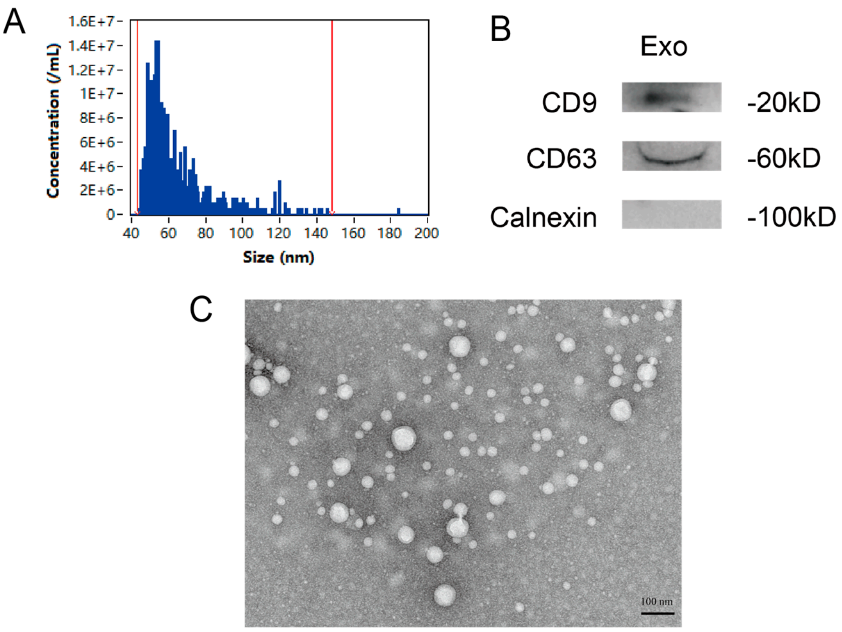

Figure S1. Characterization of miR-185 EV. (A) Particle size analysis of exosomes. (B) Western blotting for EV markers CD9, CD63 and Calnexin. (C) Representative TEM images of exosomes.

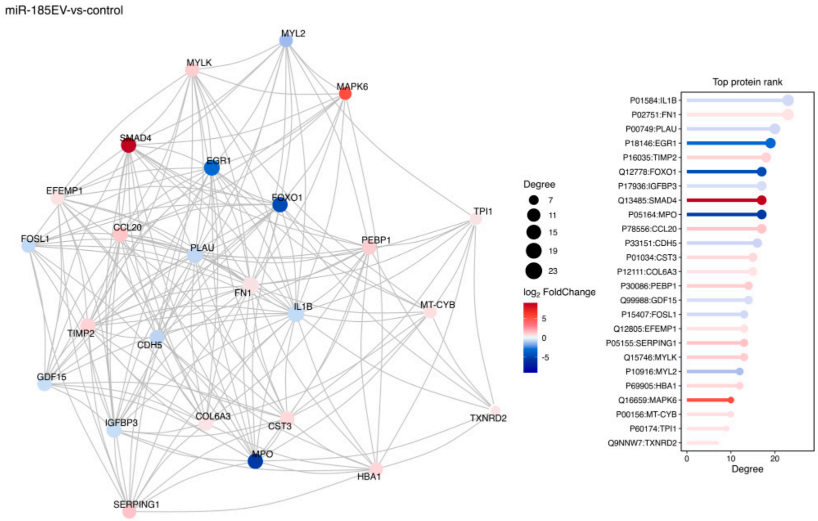

Figure S2. The PPI of the top 25 differentially expressed proteins.
